# Strengthening novice diagnostic problem solving through explicit metacognitive scaffolding in emergency medicine education

**DOI:** 10.1080/10872981.2026.2662714

**Published:** 2026-04-21

**Authors:** Chia-Yu Wang, Ming-Yuan Huang

**Affiliations:** a Graduate Institute of Digital Learning and Education, National Taiwan University of Science and Technology, Taipei, Taiwan; b Department of Emergency Medicine, MacKay Memorial Hospital, Taipei, Taiwan; c College of Medicine, MacKay Medical University, New Taipei City, Taiwan

**Keywords:** Clinical training, diagnostic reasoning, explicit instruction, medical education, metacognition, scaffolding

## Abstract

**Background:**

Metacognition is central to clinical diagnostic problem solving, yet few instructional models provide actionable guidance for embedding explicit metacognitive support into clinical training.

**Objective:**

This study aimed to introduce and evaluate the Metacognition-Integrated Instruction for Novice Diagnostic (MIND) learning model, a structured framework that integrates explicit metacognitive prompts into authentic diagnostic tasks, and to examine its impact on students’ metacognitive abilities and their structural relationships.

**Design:**

A pre–post study was conducted among 127 sixth-year medical students during a two-week emergency medicine rotation. The intervention began with a one-day MIND-based workshop, followed by repeated reinforcement of metacognitive activation in real clinical settings to support recall and transfer. Metacognitive ability was assessed using a validated Inventory of Metacognitive Self-Regulation in Diagnostic Problem Solving. Partial least squares structural equation modeling (PLS-SEM) was applied to evaluate changes in five metacognitive dimensions and their structural relationships.

**Results:**

Significant improvements were observed across all five metacognitive dimensions, with the largest gains in Objectivity and Monitoring, domains typically underdeveloped in novice diagnosticians. Structural analyses demonstrated invariance between pre- and post-intervention models, suggesting a stable underlying metacognitive structure. Knowledge of cognition and objectivity significantly predicted problem representation, which in turn predicted monitoring and evaluation. The direct path from objectivity to monitoring was not supported. No significant pre–post differences in path coefficients were identified, except for a strengthened effect of knowledge of cognition on problem representation.

**Conclusions:**

The MIND learning model provides a practice-embedded framework for strengthening diagnostic metacognition in novice physicians. By integrating explicit metacognitive scaffolds into authentic clinical encounters, the model enhances learners’ awareness of their reasoning processes and supports more effective problem framing. These findings offer actionable guidance for clinical educators seeking to incorporate metacognitive instruction into workplace-based training.

## Introduction

Diagnostic reasoning is a complex cognitive process that involves systematically applying medical knowledge and cognitive skills to resolve clinical problems while actively regulating and overseeing one’s own analytical processes in complex situations [1–5]. Metacognition often triggers a more analytic decision making [6] and significantly shapes the diagnostic problem solving by influencing how individuals approach, monitor, and evaluate their clinical reasoning [5,7], as well as how they adapt their reasoning within the next self-regulated learning (SRL) cycle, defined as learners’ active process of setting goals, monitoring performance, and adapting strategies to achieve learning outcomes [4,8]. Therefore, it is central to improving diagnostic accuracy by mitigating cognitive biases [3], which reduces diagnostic errors [9,10]. The extent to which individuals are conscious about their knowledge state, actively monitor reasoning strategies used, and evaluating accuracy of outcomes helps explain individual differences in diagnostic effectiveness despite owning similar conceptual understanding [7].

Despite years of medical education, medical students showed weaknesses in these metacognitive dimensions. Evidence shows that many medical students struggle to accurately recognise their strengths and weaknesses in learning and reasoning, often neglecting to set goals or reflect on their diagnostic performance for future improvement [7]. Novice students tend to monitor their thinking inaccurately, display overconfidence in their judgements [3,4,11], and fail to evaluate the quality or correctness of their diagnostic reasoning [7]. Some are unaware of the effectiveness of self-regulated learning (SRL) strategies and either fail to apply them when needed or discontinue them prematurely.

Although metacognition is recognised as a promising approach to mitigating cognitive biases, its consistent implementation in clinical training remains unclear [12]. In preclinical education, diagnostic reasoning is typically taught implicitly, and feedback often targets discrete outcomes rather than the underlying metacognitive processes, making it difficult to support students’ reasoning development [13]. Structured instruction and targeted strategies are therefore essential for fostering metacognitive regulation [2].

To address this gap, the study had two objectives. First, we proposed an instructional model based on SRL for teaching diagnostic problem-solving. This model operationalizes metacognition through explicit, multidimensional scaffolds tailored to emergency medicine training. We also provide guiding questions aligned with the five metacognitive dimensions to demonstrate how metacognitive prompts can be integrated into clinical diagnostic training. Second, we evaluated the model’s effectiveness in improving students’ metacognitive abilities.

### Metacognition and diagnostic reasoning

Accurate self-assessment of knowledge gaps enables learners to seek information, revise initial hypotheses, and improve diagnostic accuracy [5]. Greater metacognitive sensitivity—awareness of their diagnostic accuracy—is exhibited by pathologists who are more likely to request additional tests or second opinions when uncertain, thereby reducing errors [5]. Reflective and metacognitive engagement also helps clinicians overcome common sources of diagnostic error, such as overconfidence, premature closure, and anchoring [9,14]. Thus, metacognitive skills are essential to effective and accurate diagnostic performance [15]. Research also reported that learners with stronger metacognitive abilities demonstrate greater diagnostic accuracy, efficacy, and confidence [16,17].

### Implicit versus explicit metacognitive instruction in clinical diagnostic reasoning

Explicit and implicit approaches to teaching metacognition in diagnostic training represent two distinct pathways for developing diagnostic competence, each with distinct features and effects. In an *explicit* approach, instructors directly teach metacognitive strategies by explaining their benefits, modelling their use, and guiding application [18]. This approach views metacognition as a cognitive learning outcome, requiring students to acquire both declarative and procedural knowledge—not just what strategies to use, but when, why, and how to use them [18,19]. Classroom discussion plays a key role, helping learners construct metacognitive knowledge that is conscious and verbally accessible [19].

In contrast, an *implicit* approach involves low learner awareness, where metacognitive development occurs without direct instruction. For example, learners may engage with diagnostic tasks using prompts or forced-choice responses without being taught metacognitive strategies explicitly (e.g., [20]). The key distinction lies in the extent to which learners are provided or helped to be equipped with conceptual tools—such as knowledge about metacognition—that enable them to reflect on and discuss their diagnostic processes. Educators adopting an *implicit* approach assume that learners can develop metacognitive understanding by simply engaging in diagnostic tasks, without explicit instruction or discussion of metacognitive concepts [19], e.g., [21]. In clinical settings, metacognitive skills may emerge through observation of experienced clinicians, consistent with the principles of cognitive apprenticeship (e.g., [21]). However, while some learners develop cognitive regulation through clinical exposure, others report difficulties, particularly in planning and maintaining motivation [2].

Empirical evidence suggests *explicit* approaches are generally more effective. Compared to implicit methods, explicit instruction yields larger effect sizes in fostering metacognition [1,19]. Advocates argue that without conceptual guidance, engaging in diagnostic activities alone is unlikely to promote deep metacognitive regulation (e.g., [6]). An explicit metacognitive approach characterised by highlighting conscious, deliberate awareness of thinking processes by directly teaching metacognitive strategies (e.g., [22,23]). Explicit metacognitive instruction involves structured teaching of strategies and their benefits through curricula or scaffolds. Strategies include modelling strategy use (e.g., [24]), self-questioning with metacognitive prompts (e.g., [11]), and reflective practice, such as journaling or diagnostic checklists. In these contexts, learners are fully aware that they are acquiring metacognitive skills [20,21].

### Effectiveness of explicit metacognitive instruction in diagnostic training

Explicit metacognitive interventions have been shown to enhance students’ metacognitive awareness and knowledge, leading to improved diagnostic reasoning and reduced errors [10]. Some models have been proposed to foreground reflective and metacognitive processes in clinical reasoning, including the Contextualised Reflective Competence Framework [25], the iCARE model [26], and the Metacognitive Diagnostic Reasoning (MDR) model [1]. Despite these promising findings, explicit metacognitive instruction remains relatively uncommon in medical education, and existing frameworks vary substantially in how metacognition is conceptualised and operationalized.

For instance, the Contextualised Reflective Competence Framework [25] integrates assessment-for-learning tasks with embedded reflective elements (e.g., decision justification in the Clinical Diagnosis Assessment) to help learners identify reasoning challenges including prioritisation, management planning, and cognitive bias [13]. There is also an iCARE model that embeds regulatory strategies into the Assessment, Planning, Implementation, and Evaluation (APIE) framework, coupled with SRL-focused feedback, primarily within nursing education contexts [26]. Nursing students who engaged in iCARE reported significantly greater gains in SRL, reflective practice, clinical performance, and self-efficacy compared to those in conventional group reflection [26]. Although the results of the aforementioned studies sound promising, these approaches largely conceptualise metacognition at a broad level. These frameworks neither decompose diagnostic metacognition into distinct, teachable dimensions nor specify how instructional design can deliberately strengthen regulatory relationships among metacognitive components. Likewise, the MDR model highlights metacognition in diagnostic reasoning and acknowledges contextual and individual influences on clinicians’ thinking [1]. However, the model remains conceptual, and further empirical studies are needed to establish its feasibility.

To address the identified gaps, medical educators must foster metacognitive regulation that supports deliberate and reflective diagnostic reasoning. This requires strengthening not only individual metacognitive dimensions but also their integration. Grounded in Winne and Hadwin’s self-regulated learning (SRL) framework [27], we developed the *Metacognition-Integrated Instruction for Novice Diagnostic* (MIND) Learning model. The following sections describe the design and evaluation of a diagnostic reasoning workshop based on the MIND model intended for final-year undergraduate medical students.

## Methods

### Theoretical framework and design principles

**Figure 1.**
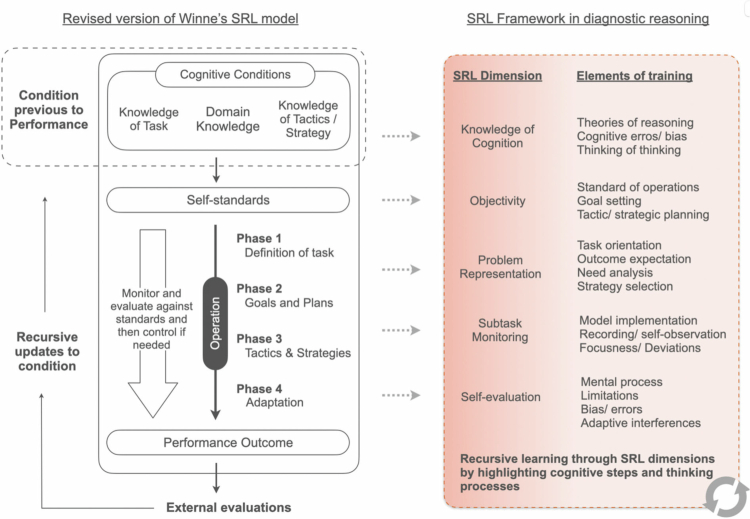
Conceptual alignment between the revised Winne and Hadwin’s SRL model and its application in diagnostic reasoning.

The MIND learning model is grounded in a revised version of Winne and Hadwin’s SRL framework [27] in the context of clinical diagnostic problem-solving. It emphasises four recursive, interdependent phases—task definition, goal setting and planning, tactics and strategies enactment, and adaptation—each influenced by cognitive conditions (task knowledge, domain knowledge, and strategic knowledge) and self-standards as internal learning goals that shape these processes (Figure 1).

In the MIND model, recursive SRL processes are mapped onto five diagnostic metacognitive dimensions (Figure 1, right): **Knowledge of Cognition, Objectivity, Problem Representation, Monitoring,** and **Evaluation**. *Knowledge of Cognition* refers to students’ awareness of diagnostic strategies, strengths, limitations, and when specific approaches are appropriate. *Objectivity* involves setting, monitoring, and revising diagnostic goals during clinical encounters. *Problem Representation* describes how learners organise and integrate clinical information into a coherent understanding of the patient’s problem. *Monitoring* captures ongoing self-regulation during diagnostic reasoning, including tracking progress, maintaining focus, and detecting deviations from intended strategies. *Evaluation* involves post-encounter reflection on reasoning quality, recognition of errors or biases, and consideration of alternative approaches. Through explicit, context-specific scaffolds embedded in authentic emergency department encounters, the model is designed to strengthen both these components and their regulatory interrelationships, making metacognitive regulation salient and actionable for novice diagnosticians.

The workshop was deliberately designed so that each dimension was explicitly operationalized through metacognitive scaffolds embedded in clinical training activities:

• **Phase 1: Definition of Task**—Students identify and interpret the diagnostic challenge presented by the patient. This requires recognising the nature of the task and accessing relevant domain knowledge. It aligns with the SRL dimension of *Knowledge of Cognition*, which includes making the diagnostic process analytical. This supports explicit understanding of cognitive biases, theories of reasoning, and the nature of expert thinking.
• **Phase 2: Goals and Plans**—Learners are guided to set diagnostic goals (e.g., identifying the most probable differential diagnoses) and plan strategic approaches (e.g., selecting question sequences or diagnostic frameworks) during diagnostic encounters. This phase links to the *Objectivity* dimension, which emphasises the importance of being mindful of mastery goals, goal clarity, operational standards, and strategic planning.
• **Phase 3: Tactics and Strategies**—Learners engage in diagnostic interviews using planned strategies. They must flexibly adapt their approach as new information emerges. This reflects the dimension of *Problem Representation*, which includes students externalising their diagnostic process, synthesising data, prioritising outcomes, and dynamically selecting enquiry strategies to differentiate between hypotheses.
• **Phase 4: Adaptation**—Based on real-time feedback and reflection, learners evaluate the success of their diagnostic approach and modify their strategies for future encounters. This aligns with both *Monitoring* and *Self-evaluation*, requiring students to identify reasoning breakdowns, recognise errors or bias, and iteratively adjust their models.

Together, these phases form a recursive feedback loop where diagnostic outcomes inform future planning by updating cognitive conditions and internal standards, cultivating adaptive, expert-like reasoning patterns.

**Figure 2.**
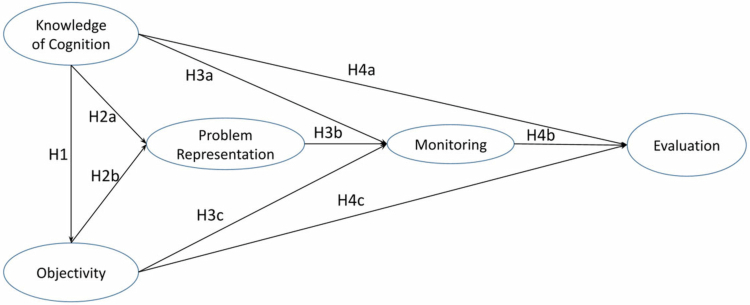
The hypothesised model that illustrates the interplay among the five dimensions of diagnostic problem solving.

In our previous study [7], the Inventory of Metacognitive Self-Regulation in Diagnostic Problem-Solving (IMSR-D) was developed to assess these five core metacognitive dimensions. The aforementioned instructional structure was informed by our validated structural model of metacognitive diagnostic reasoning [7], depicted in Figure 2). In this structural model, *Knowledge of Cognition* and *Objectivity* support *Problem Representation*, which in turn facilitates continuous *Monitoring*, leading to accurate *Evaluation* of the diagnostic outcomes. This sequential logic guided the workshop design, with sessions structured to develop each dimension of metacognitive skills while activating their interconnections in an intentional, observable sequence. These dimensions operate in a sequential and recursive structure (Figure 2), representing hypothesised directional influences in diagnostic reasoning [28]. suggest that improving knowledge of cognition and objectivity on the left side of the model may promote the development of problem representation, monitoring, and evaluation skills on the right side.

This structural model was tested among final-year undergraduate medical students [7], providing a ground for the instructional design and analysis in the present study. While the model was largely supported, participants reported low engagement across multiple dimensions, and some interconnections were missing, suggesting that metacognitive processes were insufficiently integrated [7]. These findings indicate that when instructional emphasis is placed primarily on cognitive aspects of diagnosis, novice students may develop fragmented metacognitive skills lacking the coordination required for effective diagnostic reasoning. This underscores the need for developing the MIND model. The next section explains how we applied the MIND model to design medical training with explicit metacognitive scaffolding.

### Course structure and implementation

The workshop was designed for sixth-year undergraduate medical students (UGYs) in a six-year programme, following their completion of core clinical clerkships in internal medicine, surgery, obstetrics/gynaecologist, and paediatrics. During this final year, students entered a two-week emergency medicine (EM) rotation, which serves as an integrative training period encompassing modules such as trauma, resuscitation, EMS, and toxicology. This intensive, one‐day workshop was implemented at the *entry point* serving as the *initiating scaffold* of the two-week emergency medicine rotation rather than as a brief, isolated exposure. Medical students engage continuously with real patients in small groups of three to five students per cycle and receive daily supervision from the clinical instructor. Throughout the rotation, the instructor routinely prompted students to refer back to the metacognitive goals, strategies, and monitoring checkpoints introduced during the workshop while conducting clinical interviews and formulating differential diagnoses. Thus, the metacognitive activation initiated during the workshop was repeatedly reinforced in real clinical settings over the subsequent two weeks.

**Figure 3.**
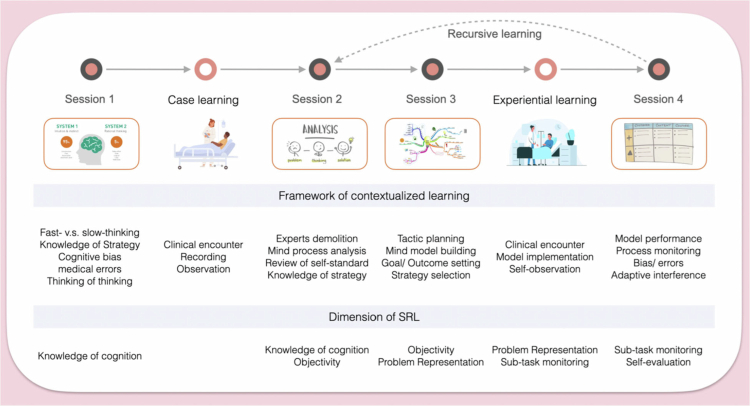
Sequenced instructional framework of the diagnostic reasoning workshop aligned with SRL dimensions.

The sessions of the workshop were structured to build on each other, aligning with SRL-based metacognitive dimensions (see Table 1 and Figure 3). Table 1 provides a detailed breakdown of the instructional content, duration, learning goals, and SRL-based metacognitive dimensions targeted in each session. A trained clinical educator facilitated each session, adopting an explicit metacognitive instructional approach. In addition to emphasising diagnostic accuracy, the instructor directed students’ attention to the metacognitive processes underlying diagnostic reasoning. These processes included awareness of one's own learning, strategy selection, goal setting, problem representation, monitoring, and evaluation. This tabular framework corresponds directly to the sequential model shown in Figure 3.

**Table 1.** Alignment of structural, objective, and targeted metacognitive dimensions and strategies in the diagnostic reasoning workshop.

Session (duration)	Instructional activity	Learning objectives	Targeted dimensions	Key strategies and tools
1. Warm-up and metacognitive reframing (60 minutes)	Pre-reading & experience sharing	Activate learners' prior knowledge and awareness of thinking modes	KoC	Group discussion; guided questions on System 1 vs 2
Case learning: authentic diagnostic encounters (30 minutes)	Real-patient interviews & observation	Establish baseline diagnostic habits; capture authentic cognitive behaviour	–	Interview recordings or written notes
2. Retrospective metacognitive scaffolding (90 minutes)	Interview behaviour analysis & introspection	Reflect on diagnostic behaviour and strategy use	KoC, Obj	Mind mapping of cognitive flow; discussion on DDx
3. Strategic reconfiguration and prospective planning (90 minutes)	Strategic reflection & problem representation	Construct diagnostic mental models based on specific symptoms	Obj, PR	Diagnostic trees, structured group discussion
Experiential learning: re-engagement with authentic patients (30 minutes)	Strategy-guided patient encounters	Apply structured strategy in real settings; develop monitoring habits	PR, Mo	Deliberate practice; guided reflection prompts
4. Integrative reflection and strategy–performance alignment (60 minutes)	Peer feedback & reflective comparison	Evaluate differences in performance; foster self-monitoring and revision	Mo, Ev	Reconstruct diagnostic diagrams; peer evaluation

Note: KoC: Knowledge of Cognition; Obj: Objectivity; PR: Problem Representations: Mo: Monitoring; Ev: Evaluation.

To support this approach, the instructor used structured teaching prompts that were explicitly aligned with the five MIND metacognitive dimensions. These prompts guided discussions and feedback during and after diagnostic encounters. The prompts targeted specific regulatory processes and were not posed ad hoc. They included strategy awareness (e.g., “What diagnostic strategy were you using here?”), goal setting and adjustment (e.g., “What was your primary goal in asking that question?”), problem representation, monitoring, and post-encounter evaluation (e.g., “What evidence supports or challenges your current differential?”). These prompts supported the real-time and retrospective articulation of reasoning by making otherwise implicit cognition explicit for structured reflection and discussion (see Appendix Table 1).

The sessions combined authentic diagnostic tasks with structured support to strengthen metacognitive regulation in response to students’ evolving needs. Table 1 aligns each session with its instructional objectives and targeted metacognitive dimensions. Appendix Table 1 provides teaching-oriented prompts for each dimension. Together, these tables provide a practical framework for implementing the MIND model in diagnostic training. The following describes how each session was implemented.

### Session 1: warm-up and metacognitive reframing

During the first session, which served as a warm-up, students shared past clinical interviews that they had found challenging or frustrating. Instead of judging diagnostic accuracy, the discussion centred on moments of uncertainty, confusion, and reasoning breakdowns. This emphasis was intended to shift the focus from the diagnosis that was reached to how thinking unfolded during the encounter. Facilitators guided students to reexamine these experiences through a metacognitive lens, using prompts such as “*Did you set explicit goals during the interview?*” and “*How might expert clinicians approach this differently?*” These questions elicited students’ knowledge of cognition (KoC) by prompting reflection on how they plan, sequence, and regulate interviews beyond medical content knowledge. Grounded in learners' authentic experiences across contexts, the session oriented students toward cognitive regulation as a core focus for diagnostic learning.

### Case learning session: authentic diagnostic encounters

Students interviewed real emergency department patients and formulated diagnostic hypotheses in the typical setting of emergency medicine, which involves time pressure, diagnostic uncertainty, and incomplete or evolving information. This session provided an authentic diagnostic experience and served as a baseline measure of students’ unscaffolded diagnostic behaviours. Conducting the encounters before providing explicit metacognitive instruction allowed us to observe students' natural questioning, hypothesis generation, and reasoning strategies. The encounters were audio-recorded to preserve the students' original diagnostic processes and were later used for individualised retrospective analysis in subsequent sessions. The clinical instructor supervised the encounters as routine clinical teaching and for patient safety, but did not otherwise intervene. All diagnostic reasoning occurred during authentic emergency department encounters. No simulated cases, standardised patients, or virtual scenarios were used.

### Session 2: retrospective metacognitive scaffolding

Audio recordings of previous student-patient interviews were used as artifacts for a retrospective metacognitive analysis. Students reviewed selected segments and created visual representations, such as mind maps or diagnostic trees, to trace how hypotheses were generated, prioritised, revised, or abandoned during real encounters. Students were also prompted to self-diagnose their reasoning by identifying uncertainty, inconsistencies, and diagnostic shortcuts and distinguishing pattern recognition from hypothetico-deductive approaches. During the feedback session, the instructor used these artifacts to help students retrace their cognitive steps, identify reasoning errors (including premature closure), and examine the alignment of their strategies, data gathering, and conclusions. These activities, framed as “*thinking about thinking*,” supported objective clarification (Obj) and deeper knowledge of cognition (KoC).

### Session 3: strategic reconfiguration and prospective planning

In this session, students shifted from retrospective analysis to prospective strategic reconfiguration. Drawing on insights from Session 2 (e.g., reasoning gaps, cognitive biases, fragmented thinking), they reconstructed their diagnostic approaches into explicit, actionable plans for future encounters. Diagnostic trees and mind maps were repurposed for planning, helping students externalise how they intended to conduct subsequent patient encounters. The instructor prompted students to set clear, actionable objectives, adopt an intentional cognitive stance (e.g., deliberate System 2 engagement), and embed self-monitoring checkpoints within their diagnostic workflow. Students also segmented the interview into stages and specified strategies for each phase. Overall, the session strengthened problem representation (PR) and monitoring-oriented regulation (Mo) of diagnosis in subsequent clinical encounters.

### Experiential learning session: re-engagement with authentic patients

After strategic planning, students re-engaged with authentic emergency department patients. Because identical cases cannot be reproduced, facilitators selected patients with similar chief complaints or symptom clusters rather than the same diagnoses. This design allowed students to apply their self-generated strategies in comparable contexts while preserving the variability and uncertainty of emergency medicine. Instruction focused on strategy enactment, including coherent questioning, structured hypothesis generation, and alignment between data gathering and planned reasoning, rather than diagnostic correctness alone. The instructor helped students sustain attention to strategic execution and metacognitive regulation during live encounters, reinforcing the application of structured diagnostic thinking under authentic conditions.

### Session 4: integrative reflection and strategy–performance alignment

In the final session, students engaged in integrative reflection by comparing their intended diagnostic strategies with their actual behaviours observed during the experiential learning phase. Referencing the visual plans developed in earlier sessions, students examined points of alignment and divergence between their planned and enacted reasoning processes. Peer feedback and facilitated discussion supported evaluation of strategy effectiveness, identification of remaining gaps, and consideration of necessary refinements. This integrative reflection emphasised metacognitive monitoring (Mo) and evaluation (Ev) by focusing on regulatory processes, not outcomes alone. Through this comparison and revision cycle, students were concluded by consolidating their metacognitive insights and preparing to adapt their diagnostic strategies in future clinical encounters.

The MIND model intentionally incorporates explicit metacognitive supports throughout all stages of diagnostic reasoning training. Rather than treating reflection and feedback as incidental, the sessions are sequenced so that students first develop cognitive awareness and goal setting, and then progress to problem representation, strategic regulation, and higher-order metacognitive processes. This design guides students through a full cycle of authentic clinical experience, retrospective reflection, prospective planning, and reengagement with diagnostic reasoning in real emergency department settings.

### Instructional design highlights

Building on the structured sequencing described above, the MIND workshop intentionally incorporated targeted metacognitive scaffolds to transform diagnostic encounters into opportunities for reflective learning. Rather than reiterating the procedural flow of each session, the design emphasised three interrelated features that operationalised the five metacognitive dimensions and strengthened their connections (see Table 1 and Figure 3).

First, metacognitive activation was achieved through explicit prompts and guided peer observation. Students began by articulating their thinking modes (System 1 versus System 2) and reflecting on prior diagnostic challenges. This made their *Knowledge of Cognition* visible, supporting clearer *goal setting*.

Second, strategic reflection was fostered through visualisation tools. Mind maps and diagnostic trees were used to externalise students’ reasoning processes, enabling them to construct and refine *problem representations* and to set purposeful *objectives*. By comparing their initial mental models with subsequent patient interviews, learners recognised gaps in logic and adjusted their strategies, thereby reinforcing *monitoring*.

Third, deliberate practice and peer feedback created recursive opportunities for repeated evaluation and adaptation. When students applied their diagnostic strategies in real patient encounters, they were encouraged to monitor their own reasoning in real time. Later, they engaged in peer critique of interview structure, comprehensiveness, and reasoning patterns. These cycles of discussion and feedback helped cultivate more accurate *evaluation* and strengthened the interplay among *knowledge of cognition, objectivity, problem representation, monitoring,* and *evaluation*.

These integrated features allowed the MIND workshop to move beyond the isolated training of single skills. The programme explicitly connected metacognitive dimensions across sequential sessions, enabling students to link their awareness, planning, and reasoning strategies with reflective evaluation. This recursive design aimed to foster adaptive, expert-like diagnostic thinking and directly address the documented gaps in novice clinicians’ metacognitive regulation.

## The present study

### Participants

All sixth-year medical students at a Medical College in Taiwan who participated in the Emergency Medicine (EM) rotation between November 11, 2020, and May 31, 2023, were invited to participate in this study. The EM rotation lasted two weeks and included multiple core modules, including trauma, resuscitation, EMS, and toxicology. These modules were delivered in small groups of three to four students. Within this broader rotation, a dedicated one-day module on diagnostic reasoning was implemented, during which the metacognitive workshop for this study took place. The study was approved by the Institutional Review Board (IRB) of MacKay Memorial Hospital, Taipei, Taiwan (IRB No. 20MMHIS484e). The requirement for written informed consent was waived by the IRB, as the study constituted an analysis of educational outcomes and posed minimal risk to participants.

This sample was obtained by recruiting consecutive cohorts over two years, reflecting the practical constraint that the annual intake of sixth-year medical students is approximately 50–65. Participation in the emergency medicine clerkship is mandatory for all final-year medical students. Therefore, all 127 eligible students attended the workshop, yielding a participation rate of 100%. Comparisons between participants and non-participants were not possible because there were no non-participants. Although the dataset included 127 cases, the paired *t*-test was computed only for the 106 participants with complete pre- and post-observations. Thus, the sample size for the pairwise comparisons is 106.

Following the suggestions of Lin et al. [29] and Ghasemy et al. [30] suggestions, we estimated the minimum required sample size using a power analysis approach recommended by [31]. With 80% statistical power and a 5% significance level, the minimum sample size was 114 participants. We analysed data from 127 participants, thereby exceeding this threshold. This sample was obtained by recruiting consecutive cohorts for more than two years, reflecting the practical constraint that the annual intake of sixth-year medical students is approximately 50–55.

A single faculty member designed and facilitated the MIND workshop, serving as the primary instructor across cohorts to ensure consistency in instructional content and pedagogical approach. While other clinical instructors participated in emergency medicine teaching activities, they did not independently deliver the MIND intervention. These instructors were part of a stable teaching team and collaborated regularly to maintain an understanding of the course’s metacognitive emphasis.

### Instruments

The Inventory of Metacognitive Self-Regulation in Diagnostic Problem-Solving (IMSR-D), developed by author [7], was designed to assess medical students’ self-reported use of metacognitive strategies in authentic diagnostic contexts. Each item of IMSR-D was phrased specifically to reflect the processes involved in clinical diagnostic reasoning. Participants were instructed to indicate how frequently they engaged in each metacognitive activity while learning or solving diagnostic problems by responding on a 5-point Likert scale (1 = never, 2 = rarely, 3 = sometimes, 4 = often, 5 = always). Higher scores reflected stronger metacognitive regulation relevant to diagnostic tasks.

### Data collection

The diagnostic reasoning workshop was implemented on the first day of each student group's two-week emergency medicine rotation. At the start of Session 1, students took a pretest on their personal devices using the IMSR-D instrument via Google Forms. During the “Case Learning” activity and Session 2, diagnostic interviews were audio-recorded with patient consent to support subsequent analysis of students’ reasoning patterns. Audio recordings of student-patient interviews were only used to support retrospective metacognitive reflection and guided discussion. The recordings were not analysed as research data, and no outcomes were derived from them. All recordings were deleted after the teaching sessions. Collaborative diagnostic tasks using the online whiteboard platform Miro (San Francisco, California, USA) were the focus of Sessions 3 and 4. This facilitated shared modelling and strategic reflection. At the end of the two-week EM rotation, students took a posttest using the same IMSR-D instrument and administration method.

### Data analysis

To evaluate the intervention’s effectiveness, we conducted paired-sample t-tests to examine the gain of metacognitive regulatory skills from the pretest to posttest. Pre-post comparisons of structural models were also performed, utilising a commercial software, Smart-PLS version 4.1.1, for partial least squares structural equation modelling (PLS-SEM). These analyses assessed improvements in individual metacognitive competencies, as well as changes in the structural relations among metacognitive components following the workshop. PLS-SEM is a variance-based approach [32]. It can accommodate non-normally distributed data using a nonparametric bootstrapping procedure and is typically more tolerant of smaller sample sizes than covariance-based structural equation modelling (CB-SEM) [31]. Accordingly, PLS-SEM has been widely applied in digital learning and higher education research (e.g., review studies by [29,30]).

### Evaluation of PLS-SEM models

Following the suggestions from [29], we evaluated the PLS-SEM results using a two-stage procedure: measurement (outer) model evaluation and structural (inner) model evaluation. To test the significance of the hypothesised relationships, we applied nonparametric bootstrapping with 5,000 resamples to obtain the path estimates and their corresponding *p*-values. Structural modelling was conducted using item scores from the pretest and the posttest of IMSR-D.

For the *measurement (outer) model evaluation*, *indicator reliability* was examined via factor loadings, with values above 0.70 considered acceptable, while items with loadings below 0.40 were excluded [33]. *Internal consistency* reliability was assessed using composite reliability (CR). All CR values exceeded the recommended threshold of 0.70, suggesting that the items collectively measure a coherent construct [34]. *Convergent validity* was evaluated through the average variance extracted (AVE), computed as the mean of squared factor loadings for items within the same construct. An AVE value exceeding 0.50 is generally considered satisfactory [34]. To determine *discriminant validity*, the *Fornell-Larcker criterion* was applied. According to this standard, the square root of each construct’s AVE should be greater than its correlations with other constructs in the model [34].

Table 2 presents the results of the measurement model validation for the pretest and posttest of IMSR-D, summarising the factor loadings, composite reliability (CR), and average variance extracted (AVE) for each dimension. The majority of items demonstrated factor loadings exceeding the recommended threshold of 0.70. A few items fell within the 0.63 to 0.69 range but were retained, as other indicators within the same construct met acceptable standards [33]. Descriptive statistics for pre- and post-test items are also reported in Table 2.

**Table 2.** Results of the outer model validation for the pretest and posttest of IMSR-D.

		Pre					Post					
Dimensions	Items	Mean (SD)	factor loading	CR	AVE	Cronbach’s α	Mean (SD)	factor loading	CR	AVE	Cronbach’s α	
KoC				0.68	0.51	0.68			0.75	0.56	0.74
	KoC1	3.07 (0.99)	0.66				3.74 (0.76)	0.70			
	KoC2	3.72 (0.96)	0.63				4.33 (0.65)	0.77			
	KoC3	2.61 (0.97)	0.81				3.68 (0.76)	0.81			
	KoC5	3.17 (0.96)	0.74				3.86 (0.70)	0.71			
Obj				0.79	0.62	0.79			0.74	0.56	0.74
	Obj1	3.41 (1.07)	0.76				4.20 (0.67)	0.78			
	Obj2	3.63 (1.05)	0.81				4.11 (0.69)	0.76			
	Obj3	3.25 (1.12)	0.75				4.14 (0.68)	0.76			
	Obj4	3.32 (1.15)	0.82				4.05 (0.82)	0.68			
PR				0.75	0.48	0.74			0.79	0.55	0.79
	PR1	4.10 (0.74)	0.68				4.38 (0.65)	0.74			
	PR3	3.40 (0.90)	0.66				3.96 (0.74)	0.73			
	PR4	3.40 (0.88)	0.66				4.08 (0.66)	0.73			
	PR5	3.67 (0.91)	0.72				4.30 (0.71)	0.74			
	PR6	3.85 (0.86)	0.76				4.29 (0.65)	0.75			
Mo				0.77	0.52	0.77			0.81	0.56	0.81
	Mo2	3.74 (0.82)	0.68				4.19 (0.71)	0.78			
	Mo4	3.34 (0.95)	0.73				3.93 (0.78)	0.66			
	Mo6	2.90 (0.87)	0.78				3.77 (0.76)	0.72			
	Mo7	2.78 (0.86)	0.72				3.86 (0.71)	0.81			
	Mo8	3.88 (0.79)	0.69				4.25 (0.68)	0.78			
Ev				0.78	0.53	0.77			0.81	0.55	0.80
	Ev1	3.36 (1.02)	0.77				4.14 (0.74)	0.73			
	Ev2	3.64 (0.89)	0.75				4.19 (0.61)	0.83			
	Ev3	3.40 (0.86)	0.73				4.13 (0.74)	0.82			
	Ev4	3.40 (0.86)	0.73				3.84 (0.80)	0.66			
	Ev5	3.78 (0.77)	0.74				4.13 (0.69)	0.67			

Note: Items removed because factor loadings below 0.40. Ten items were removed including Obj5, Obj6, Obj7, PR2, Mo1, Mo3, Mo5, Mo9, Ev6, and Ev7.

Regarding *internal consistency*, CR values for all dimensions ranged from 0.68 to 0.82 (see also Table 2), surpassing the minimum criterion of 0.70. *Convergent validity* was supported by AVE values between 0.48 and 0.62; all were above the 0.50 benchmark, except the CR value for PR, which was slightly lower than the conventional criterion at 0.48. As evaluated by Cronbach's *α*, the *reliability* estimates for each dimension were acceptable for both the pre- and post-tests (pretest: Knowledge of Cognition = .68, Objectivity = .79, Problem Representation = .74, Monitoring = .77, and Evaluation = .78. For the posttest, the values were as follows: Knowledge of Cognition = .75, Objectivity = .74, Problem Representation = .79, Monitoring = .81, Evaluation = .81). These results meet the suggested criterion [33].

To assess *discriminant validity*, the square root of the AVE, as shown along the diagonal for each construct, was compared to the correlations among the dimensions for the pre- and post-tests (Table 3). In line with the Fornell-Larcker criterion, the square root of each AVE (ranging from 0.71 to 0.79) exceeded the corresponding Pearson correlation coefficients, indicating adequate discriminant validity. All dimensions demonstrated significant correlations at the moderate levels (*r* = 0.54–0.69 for pretest and r = 0.55–0.72 for posttest), supporting both conceptual distinctiveness and structural coherence. Overall, the internal consistency, convergent validity, and discriminant validity of both pre-and post-tests met the criteria suggested by [33].

**Table 3.** The correlations and discriminant validity among the dimensions of pre- and Post-tests IMSR-D.

					Pre				Post	
Dimensions	KoC	Obj	PR	Mo	Ev	KoC	Obj	PR	Mo	Ev
KoC	**0.71**					**0.75**				
Obj	0.57	**0.79**				0.64	**0.75**			
PR	0.56	0.63	**0.70**			0.70	0.64	**0.74**		
Mo	0.68	0.55	0.70	**0.72**		0.64	0.55	0.72	**0.75**	
Ev	0.64	0.68	0.65	0.68	**0.73**	0.67	0.70	0.75	0.73	**0.74**

The indexes and results of the *structural (inner) model evaluation* of the model were examined and reported in the Findings section. We then compared the structural models before and after the MIND intervention. To determine if a path coefficient changes significantly between time points, we compared the pretest PLS-SEM models across time using the parametric PLS-multigroup analysis (MGA) [35]. A three-step process, suggested by [36], was used to analyse measurement invariance of composite models (MICOM) before performing the PLS-MGA [35]. Differences in path coefficients across time were tested following established procedures (e.g., [35,37]).

### Findings

#### Effects of the explicit metacognitive MIND learning model

To examine the effectiveness of the MIND learning model in enhancing undergraduate medical students’ metacognitive regulation in diagnostic problem-solving, paired-sample *t*-tests were conducted on pre- and post-test scores across the five IMSR-D dimensions. As shown in Table 4, students initially reported relatively low metacognitive competence, with mean scores ranging from 3.11 to 3.63. After the intervention, all five dimensions showed statistically significant improvement, with posttest means between 3.91 and 4.16. The gains were accompanied by large effect sizes across all dimensions (Cohen’s *d* > 0.98).

**Table 4.** Descriptive and Inferential Statistics for Pre- and Post-Test Scores on IMSR-D (*n* = 106).

Metacognitive dimensions		Pre		Post		Mean diff		*t*	Cohen’s *d*
	Items	*M*	*SD*	*M*	*SD*	*M*	*SD*		
Knowledge of cognition	4	3.11	.71	3.91	.54	.80	.64	12.86***	1.27
Objectivity	6	3.16	.72	4.02	.52	.86	.61	14.38***	1.37
Problem representation	6	3.63	.57	4.16	.51	.53	.58	9.44***	0.98
Monitoring	9	3.33	.59	4.03	.49	.70	.55	13.10***	1.29
Evaluation	7	3.30	.62	4.04	.46	.74	.63	12.19***	1.36

Note: ***: *p* < .001.

After the workshop, the participants reported higher diagnostic metacognition across all five dimensions, including greater self-awareness of their cognitive processes and learning goals, improved problem structuring and clarification, stronger monitoring of diagnostic reasoning, and more conscious outcome evaluation. Notably, the largest gains were observed in *Knowledge of Cognition*, *Objectivity, Monitoring,* and *Evaluation* (*d* = 1.27, 1.37, 1.29, and 1.36, respectively), dimensions that are often underdeveloped in novice diagnosticians.

#### The structural model evaluation and comparisons of the model before and after the MIND intervention

For the *structural (inner) model evaluation* of the model, we interpreted bootstrapped path coefficients and *p*-values, and evaluated predictive performance using the coefficient of determination (*R²*) [31]. The *R*² value represents the proportion of variance in an endogenous construct explained by its predictors; higher *R*² values indicate stronger predictive performance in PLS-SEM [31].

**Figure 4.**
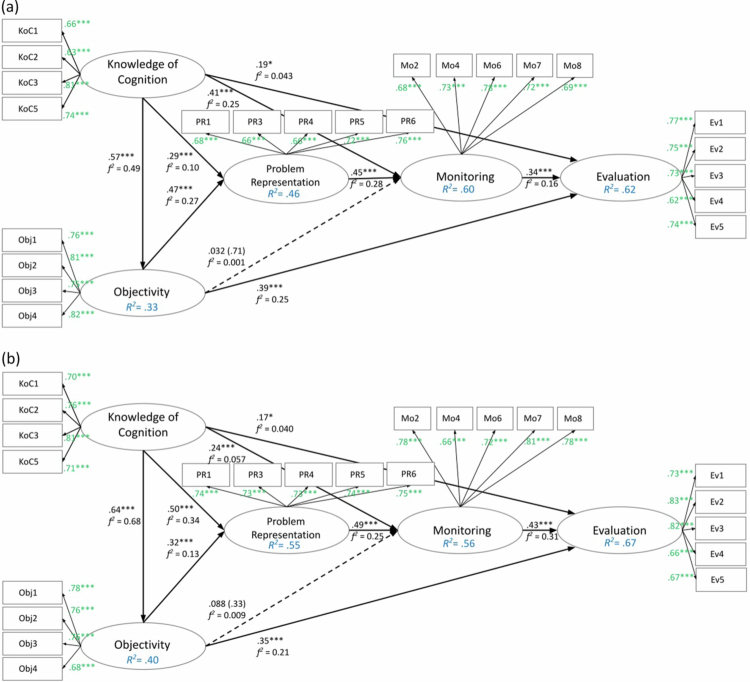
a. The results of the structural relationships examination of pretest IMSR-D (*n* = 127). Note: ***: *p* < 0.01; Non-significant paths were indicated as dashed lines. b.The results of the structural relationships examination of posttest IMSR-D (*n* = 127). Note: *: *p* < 0.1; ***: *p* < 0.01, as suggested by [38,39]; Non-significant paths were indicated as dashed lines.

The output of the KoC, Obj, PR, Mo, and Ev dimensions for the pretest and posttest models evaluation, including loadings and path coefficients in PLS-SEM software is shown in Figure 4a and b. In these models, the numbers inside the ovals show the *R²* values and the bold numbers are related to the posttest PLS-SEM models. Tables 5 and 6 present a summary of the results from examining model comparisons for the path coefficients and for *R²* over time.

**Table 5.** Path coefficient for pretest and posttest models and *p*-values for MGA test statistics.

	Pre	Post	
Construct relationships	*β*	*β*	*p*
KoC → Obj	.57***	.64***	.40
KoC → PR	.29***	.50***	.093
KoC → Mo	.41***	.24***	.19
KoC → Ev	.19*	.17*	.87
Obj → PR	.47***	.32***	.25
Obj → Mo	.032	.088	.67
Obj → Ev	.39***	.35***	.75
PR → Mo	.45***	.49***	.72
Mo → Ev	.34***	.43***	.44

Note: *: *p* < .1; ***: *p* < .01.

**Table 6.** *R*^*2*^ for pretest and posttest models and *p*-values for MGA test statistics.

	Pre		Post		
Dimensions	*R* ^*2*^	GoF	*R* ^*2*^	GoF	*p*
Obj	.33	.45	.40	.47	.47
PR	.46	.47	.55	.55	.30
Mo	.60	.56	.56	.56	.61
Ev	.62	.57	.67	.61	.47

Non-significant paths were indicated as dashed lines for clarity. Figure 4a and b display the significant path coefficients, *t*-values, and *R²* values. Across both models, several consistent relationships emerged. Students’ *Knowledge of Cognition* significantly predicted their *Objectivity*. Together, *Knowledge of Cognition* and *Objectivity* significantly predicted *Problem Representation*. *Monitoring* was predicted by both *Knowledge of Cognition* and *Problem Representation*. *Evaluation* was significantly predicted by *Knowledge of Cognition,* *Objectivity*, and *Monitoring*. Of the hypothesised structural relations, only the path from *Objectivity* to *Monitoring* was non-significant in both models.

According to Table 5, the result from path coefficient comparisons over time indicated that the structural path from KoC to PR strengthened significantly (*p* < 0.1), increasing from *β* = .29 (pretest) to *β* = .50 (posttest). Other path coefficients remain stable and were insignificant prior to and after the MIND-based intervention.

The *R²* values in Table 6 and Figure 4a and b indicate that the explained variance ranges from moderate to substantial levels across the constructs in the pretest and posttest models, according to [40] guidelines (0.67 is substantial, 0.33 is moderate, and 0.19 is weak). While the Goodness-of-Fit (GoF) index is not suitable for validating the PLS-SEM model [41], it can be used to evaluate the predictive power across different datasets. The GoF index ranges from 0 to 1, where 0.1 (small), 0.25 (moderate), and > 0.36 (large) are benchmarks. All *R²* values reported in Table 6 exceed the criteria. While some constructs showed notable changes in explained variance (e.g., PR increased from 46% to 55%), these changes did not reach statistical significance. No significant differences were detected for any of the four endogenous constructs across time, although the path from KoC to PR was strengthened after the intervention.

## Discussion

### Effectiveness of explicit metacognitive scaffolding

Consistent with evidence that metacognitive sensitivity and reflective engagement support diagnostic accuracy and mitigate cognitive biases [5,10,11,13–16,22,27], the MIND workshop produced significant improvements across all IMSR-D dimensions, with the largest gains in *Objectivity* and *Monitoring*, areas often underdeveloped in novice diagnosticians. Although adopting new strategies requires greater effort, explicit instruction provides clear frameworks for metacognitive processes, enabling students to allocate cognitive resources to link between metacognitive components [18]. Meanwhile, the instructor made the metacognitive knowledge explicit through explicit explanation, modeling when and how to use strategies [42], raising their awareness of the benefit of strategy use, and motivating learners to apply them in practice. Through prompts, visualisation tools for externalising thought, and structured peer evaluation, scaffolds transformed abstract metacognitive concepts into actionable routines for clinical interviewing and reasoning.

Although metacognition was assessed solely via self-report, well-designed items can capture learners’ generalised perceptions of metacognitive activities across prior learning events [43]. The IMSR-D, adapted from the IMSR [44], was developed to represent multiple metacognitive skills relevant to problem solving. Prior work by the first author [45] applied the same five-construct inventory in chemistry problem solving and found moderate, significant associations between self-report scores and observed metacognitive behaviours coded from think-aloud protocols. Thus, while self-report primarily reflects perceived metacognition, it remains a meaningful indicator of metacognitive skills.

This study presents the results of a single-group pre- and post-test design and lacks a control group. This makes it impossible to clarify the effect of metacognitive scaffolding. However, by proposing metacognitive prompts for clinical diagnostic training that clearly correspond to the metacognitive regulatory theory, we believe that these findings support prior claims that explicit instruction benefits novice learners and produces broad improvements across all five dimensions [6,11,13,46]. Future research should design rigorous quasi-experimental studies to compare the effectiveness of explicit and implicit metacognitive scaffolding.

### Comparisons of the structural model across time

This pattern of structural invariance, as revealed in the stable relational structure and most of the path coefficients, suggests the stability of the overall structural model in explaining development of metacognitive regulation in diagnostic problem-solving across the intervention. This finding implies that the validated theoretical model from the authors' previous work remained stable. The amount of variance in downstream metacognitive dimensions explained by the upstream components KoC and Obj remains stable over time [28]. Our findings can be interpreted through existing learning theories. Explicit instruction reflects the principles of the cognitive apprenticeship model by making otherwise implicit reasoning processes visible to learners [42]. Explicit support and instruction foster coherent metacognitive structures rather than isolated skills and increase network connectivity from pretest to posttest [47].

Despite the explicit focus of the MIND workshop on goal-setting and monitoring, the regulatory link from *Objectivity* to *Monitoring* was not observed within the two-week timeframe. This persistent null finding suggests that not all metacognitive skills respond equally to brief, explicit instruction. The enactment of goal-directed monitoring during high-stakes clinical encounters may require additional pedagogical support. Linking stated intentions into in-the-moment monitoring under cognitive demand, time pressure, and diagnostic uncertainty, conditions inherent to emergency medicine, likely requires deliberate clinical practice and sustained or iterative support.

The path coefficient comparisons show a significant strengthening on the path from KoC to PR. This finding implies that the MIND intervention enhances students’ ability to apply their knowledge of cognition to create more coherent clinical problem representations. As students become more aware of their diagnostic reasoning, they may be better in organising and integrating clinical information into structured formulations. The workshop made use of visualisation tools (e.g., diagnostic trees and mind maps) and retrospective analysis. These scaffolds likely made the connection between awareness and representation explicit and actionable. This aligns with Winne's [27] SRL framework, which highlights the central role of knowledge of cognition, including declarative strategic knowledge with knowledge of when and how to apply them, as an essential precondition for other regulatory processes in diagnostic reasoning [48].

For novice practitioners, engaging in cycles of reflection supports adaptation [49] by helping them understand the goals of the task [50], aware of their own objectives, and develop awareness of how to learn for more accurate diagnosis and preventing errors [49] through monitoring and evaluation over time. This process is particularly important in the early years of training.

### Conclusions, implications, and limitations

This study offers both practical and theoretical contributions to medical education. By embedding explicit metacognitive scaffolds within authentic diagnostic encounters, the MIND workshop operationalised regulatory processes that prior frameworks largely described conceptually [1,25]. The contribution of this study lies in treating the metacognition process in diagnostic reasoning as multidimensional and developing the IMSR-D questionnaire based on theoretical definitions to measure these five dimensions. Previous discussions on the importance of metacognition in diagnostic problem solving such as the MDR model [1] have focused on presenting theoretical stances without providing empirical evidence. These discussions such as iCARE [26] have also assessed metacognition as a whole (e.g., treating reflection as metacognition) and have failed to examine the possible connections between these constructs or to use multidimensional measurement.

In this study, we developed a model based on Winne's theory and computational thinking problem-solving theory to explore the structural relations of the five constructs. Furthermore, we propose a clear pedagogical design, with corresponding guiding questions for educators interested in adapting the MIND model. With PLS-MGA analysis, our findings also strengthen inference beyond simple pre–post comparisons and support structural invariance of the five-dimensional IMSR-D model, capturing interactions that may be overlooked by conventional analyses [51].

It is important to note that these findings should be interpreted in light of the intervention's instructional intent. The MIND workshop was designed to initiate, rather than complete, students’ metacognitive development. Subsequent clinical encounters serve as the primary context for enactment and consolidation. Thus, the observed changes reflect the initial activation and organisation of metacognitive regulation rather than mastery or long-term diagnostic competence. This distinction is important when considering the implications and limitations of the present study. The persistently non-significant path from Objectivity to Monitoring is therefore informative, suggesting differential responses for the acquisition of metacognitive skills. Future studies should test enhanced pedagogical supports targeting this pathway (e.g., more explicit instruction and deliberate practice) and compare strengthened versus standard MIND implementations using PLS-MGA.

Several limitations should be acknowledged. First, the intervention was delivered as a one-day workshop within an emergency medicine rotation. Although this approach is effective at initiating metacognitive awareness and restructuring regulatory relations, metacognitive development and its application to new clinical situations usually necessitate multiple cycles of enactment and reflection. Therefore, future studies should examine longitudinal reinforcement designs that include repeated, low-dose metacognitive prompting and the gradual fading of instructional scaffolds to better understand how metacognitive regulation stabilises and becomes internalised over time.

Second, the feasibility and sustainability of this intervention depend on local educational resources and faculty preparedness. In the present study, a designated teaching physician supported implementation, focusing primarily on educational facilitation rather than direct clinical service. While this arrangement may not be broadly accessible in emergency medicine settings, the core instructional principles of the MIND model, such as explicit and structured metacognitive prompting of reasoning processes, can be adapted to different resource contexts. Future research should examine how faculty development in metacognitive instruction influences scalability across institutions.

Third, this study lacked a control group. This design choice was influenced by the ethical constraints of the required emergency medicine clerkship, which prohibited withholding an educational intervention from a subset of students. Furthermore, emergency department training entails highly varied patient presentations and shared clinical teaching environments. This increases the risk of contamination between groups and complicates between-group comparisons. Accordingly, we adopted a pre-post within-subject design and interpreted the findings cautiously, avoiding causal claims. Future studies using quasi-experimental or longitudinal designs may strengthen causal inference when ethical and contextual constraints permit.

Finally, metacognitive regulation was primarily assessed through self-report and process data were not collected to supplement the explanation of the behavioural changes that occurred. We acknowledge that the absence of performance-based measures limits our ability to draw conclusions about the translation of metacognitive gains into observable diagnostic behaviours. However, assessing diagnostic performance in the emergency department is challenging due to patient heterogeneity, time constraints, and the brief duration of undergraduate clinical rotations. Additionally, short-term performance metrics may not adequately reflect the development of metacognitive regulation, which typically involves iterative reflection and strategy adaptation over time rather than immediate improvements in diagnostic accuracy. Although PLS-SEM allows us to detect structural changes in the relationships among dimensions, the correlational design cannot establish causality. Future research should triangulate self-report data with behavioural or performance-based indicators of diagnostic reasoning, and where feasible, employ quasi-experimental or longitudinal designs to strengthen causal inference.

## Data Availability

The datasets generated and/or analysed during the current study are available from the corresponding author upon reasonable request.

## Appendix

**Table 1.** MIND-aligned metacognitive prompts for diagnostic interviewing and clinical problem solving (teaching-oriented version)

MIND demension	Instructional focus	Teaching prompts (examples in diagnostic interviewing)
**Knowledge of cognition (KoC)**	Awareness of reasoning strategies, triggers, and limitations	What made you think of this diagnosis first—was it more intuition, experience, or deliberate reasoning? What diagnostic strategy are you using right now? When does this approach usually work well, and when might it mislead you? In similar future cases, how would you intentionally change your interviewing approach?
**Objectivity (Obj)**	Goal setting and goal adjustment during the interview	What was your main goal when you started this interview? Did any new information make you change that goal during the interview? Looking back, did this interview help you achieve your original goal? How would you set a clearer or more actionable goal next time?
**Problem representation (PR)**	Structuring and synthesising the clinical problem	Can you summarise this patient’s core problem in one sentence? Do you feel all of the patient’s problems have been fully captured? What is still unclear? Do you have enough information to judge severity and prioritise problems? Are the cause–effect links you are assuming reasonable, or do they need further checking?
**Monitoring (Mo)**	Tracking reasoning processes during information gathering	Are your current questions still aligned with your original diagnostic goal? Do you notice that you may be narrowing down too quickly on one diagnosis? Are there signals suggesting that you need to slow down, ask follow-up questions, or change strategy? Could this line of questioning cause you to miss other important diagnoses?
**Evaluation (Ev)**	Reflecting on reasoning quality and future improvement	Looking back, which diagnoses were not sufficiently explored? Which part of your reasoning was most vulnerable to bias or premature closure? If you could do this interview again, what would you do differently? What does this case remind you to pay attention to next time you see a similar patient?

## References

[1] Beebe SL, McNelis AM, El-Banna M, et al. Reflecting on diagnosis: the metacognitive diagnostic reasoning model ©. J Am Assoc Nurse Pract 2024 Dec;36(12):711–718. doi: 10.1097/JXX.0000000000001018 39631043

[2] Ruth AA, Dzara K. Incorporating structured metacognitive training into an undergraduate anatomy classroom. Anat Sci Educ. 2025;18(1):87–96. doi: 10.1002/ase.2537 39586835

[3] Wang T, Zheng J, Tan C, et al. Computer-based scaffoldings influence students’ metacognitive monitoring and problem-solving efficiency in an intelligent tutoring system. J Comput Assist Learn. 2023;39(5):1652–1665. doi: 10.1111/jcal.12824

[4] von Hoyer J, Bientzle M, Cress U, et al. False certainty in the acquisition of anatomical and physiotherapeutic knowledge. BMC Med Educ. 2022 Nov;22(1):765. doi: 10.1186/s12909-022-03820-x 36348330 PMC9641864

[5] Clayton DA, Eguchi MM, Kerr KF, et al. Are pathologists self-aware of their diagnostic accuracy? Metacognition and the diagnostic process in pathology. Med Decis Mak Int J Soc Med Decis Mak 2023 Feb;43(2):164–174. doi: 10.1177/0272989X221126528 PMC9825636 36124966

[6] Royce CS, Hayes MM, Schwartzstein RM. Teaching critical thinking: a case for instruction in cognitive biases to reduce diagnostic errors and improve patient safety. Acad Med J Assoc Am Med Coll 2019 Feb;94(2):187–194. doi: 10.1097/ACM.0000000000002518 30398993

[7] Wang C-Y, Chen S, Huang M-Y. Exploring medical students’ metacognitive and regulatory dimensions of diagnostic problem solving. Med Educ Online. 2023 Dec;28(1):2210804. doi: 10.1080/10872981.2023.2210804 37198958 PMC10198001

[8] Hartjes MG, Richir MC, Cazaubon Y, et al. Enhancing therapeutic reasoning: key insights and recommendations for education in prescribing. BMC Med Educ. 2024 Nov;24(1):1360. doi: 10.1186/s12909-024-06310-4 39587582 PMC11590475

[9] Vally ZI, Khammissa RAG, Feller G, et al. Errors in clinical diagnosis: a narrative review. J Int Med Res. 2023 Aug;51(8):3000605231162798. doi: 10.1177/03000605231162798 37602466 PMC10467407

[10] Beebe SL, McNelis AM, El-Banna M, et al. Nailing the diagnosis: using screen-based simulation to improve factors of diagnostic reasoning in family nurse practitioner education. Clin Simul Nurs. 2024 June;91:101528. doi: 10.1016/j.ecns.2024.101528

[11] Garbayo LS, Harris DM, Fiore SM, et al. A metacognitive confidence calibration (MCC) tool to help medical students scaffold diagnostic reasoning in decision-making during high-fidelity patient simulations. Adv Physiol Educ. 2023 Mar;47(1):71–81. doi: 10.1152/advan.00156.2021 35981722

[12] Yesudian RI, Yesudian PD. A new model for categorizing cognitive biases and debiasing strategies in dermatology. Int J Dermatol. 2023 Feb;62(2):137–142. doi: 10.1111/ijd.16348 35802380

[13] Ainge LE, Edgar AK, Kirkman JM, et al. Developing clinical reasoning along the cognitive continuum: a mixed methods evaluation of a novel clinical diagnosis assessment. BMC Med Educ. 2025 Jan;25(1):31. doi: 10.1186/s12909-024-06613-6 39780160 PMC11708177

[14] Jaspan V, Schaye V, Parsons AS, et al. Lessons in clinical reasoning ‒ pitfalls, myths and pearls: a case of recurrent pancreatitis. Diagn Berl Ger 2021 Dec;9(2):288–293. doi: 10.1515/dx-2021-0035 34882358

[15] Zheng J, Li S, Lajoie SP. Diagnosing virtual patients in a technology-rich learning environment: a sequential mining of students’ efficiency and behavioral patterns. Educ Inf Technol 2022 Apr;27(3):4259–4275. doi: 10.1007/s10639-021-10772-0

[16] Zheng J, Lajoie SP, Wang T, et al. Supporting self-regulated learning in clinical problem-solving with a computer-based learning environment: the effectiveness of scaffolds. Metacognition Learn. 2023 Dec;18(3):693–709. doi: 10.1007/s11409-023-09352-z

[17] Lajoie SP, Li S, Zheng J. The functional roles of metacognitive judgement and emotion in predicting clinical reasoning performance with a computer simulated environment. Interact Learn Environ. 2023 Aug;31(6):3464–3475. doi: 10.1080/10494820.2021.1931347

[18] Dignath C, Veenman MVJ. The role of direct strategy instruction and indirect activation of self-regulated Learning—Evidence from classroom observation studies. Educ Psychol Rev. 2021 June;33(2):489–533. doi: 10.1007/s10648-020-09534-0

[19] Abd-El-Khalick F, Lederman NG. Improving science teachers’ conceptions of nature of science: a critical review of the literature. Int J Sci Educ. 2000 July;22(7):665–701. doi: 10.1080/09500690050044044

[20] Tanoue H, Yoshinaga N, Hayashi Y, et al. Clinical effectiveness of metacognitive training as a transdiagnostic program in routine clinical settings: a prospective, multicenter, single-group study. Jpn J Nurs Sci. 2021;18(2):e12389. doi: 10.1111/jjns.12389 33174673

[21] Salajegheh M, Rooholamini A, Norouzi A. Investigating the role of clinical exposure on motivational self-regulation skills in medical students based on cognitive apprenticeship model. BMC Med Educ. 2024 Mar;24(1):257. doi: 10.1186/s12909-024-05253-0 38459546 PMC10921607

[22] Hsu C-W. Mind over prejudice: an implicit bias training in medical education using cognitive bias modification. J Grad Med Educ. 2023 Oct;15(5):541–543. doi: 10.4300/JGME-D-23-00146.1 37781427 PMC10539152

[23] Pikouli FA, Moraitou D, Papantoniou G, et al. Metacognitive strategy training improves decision-making abilities in amnestic mild cognitive impairment. J Intell. 2023 Sept;11(9):182. doi: 10.3390/jintelligence11090182 37754911 PMC10532678

[24] Wang C, Li J, Xia Y, et al. Learning from errors? The impact of erroneous example elaboration on learning outcomes of medical statistics in Chinese medical students. BMC Med Educ. 2022 June;22(1):469. doi: 10.1186/s12909-022-03460-1 35710473 PMC9203230

[25] Lane AS, Roberts C. Contextualised reflective competence: a new learning model promoting reflective practice for clinical training. BMC Med Educ. 2022 Jan;22(1):71. doi: 10.1186/s12909-022-03112-4 35093060 PMC8801113

[26] Zeng W, Goh YX, Ponnamperuma G, et al. Promotion of self-regulated learning through internalization of critical thinking, assessment and reflection to empower learning (iCARE): a quasi-experimental study. Nurse Educ Today. 2024 Nov;142:106339. doi: 10.1016/j.nedt.2024.106339 39151387

[27] Winne PH, Hadwin AF. Studying as self-regulated learning In: Metacognition in educational theory and practice, in The educational psychology series. Mahwah, NJ, US: Lawrence Erlbaum Associates Publishers; 1998. pp. 277–304.

[28] Tsai M-J, Liang J-C, Lee SW-Y, et al. Structural validation for the developmental model of computational thinking. J Educ Comput Res. 2022 Mar;60(1):56–73. doi: 10.1177/07356331211017794

[29] Lin H-M, Lee M-H, Liang J-C, et al. A review of using partial least square structural equation modeling in e-learning research. Br J Educ Technol. 2020;51(4):1354–1372. doi: 10.1111/bjet.12890

[30] Ghasemy M, Teeroovengadum V, Becker J-M, et al. This fast car can move faster: a review of PLS-SEM application in higher education research. High Educ. 2020 Dec;80(6):1121–1152. doi: 10.1007/s10734-020-00534-1

[31] Hair J, Hult GTM, Ringle C, et al. Eds In: A Primer on Partial Least Squares Structural Equation Modeling. 2nd ed. Thousand Oaks: Sage; 2017.

[32] Jöreskog KG, Wold HOA. The ML and PLS techniques for modeling with latent variables: historical and comparative aspects. Systems Under Indirect Observation: Causality, Structure, Prediction, in Part I. Amsterdam: Elsevier; 1982. p. 263–270.

[33] Hair JF, Risher JJ, Sarstedt M, et al. When to use and how to report the results of PLS-SEM. Eur Bus Rev. 2019 Jan;31(1):2–24. doi: 10.1108/EBR-11-2018-0203

[34] Sarstedt JF. Factors versus composites: guidelines for choosing the right structural equation modeling method. Proj Manag J. 2019 Dec;50(6):619–624. doi: 10.1177/8756972819882132

[35] Roemer E. A tutorial on the use of PLS path modeling in longitudinal studies. Ind Manag Data Syst. 2016 Oct;116(9):1901–1921. doi: 10.1108/IMDS-07-2015-0317

[36] Henseler J, Ringle CM, Sarstedt M. Testing measurement invariance of composites using partial least squares. Int Mark Rev. 2016 May;33(3):405–431. doi: 10.1108/IMR-09-2014-0304

[37] Mohammadi RR, Saeidi M, Ahangari S. Self-regulated learning instruction and the relationships among self-regulation, Reading comprehension and Reading problem solving: PLS-SEM approach. Cogent Educ. 2020 Jan;7(1):1746105. doi: 10.1080/2331186X.2020.1746105

[38] Chang H-Y, Liang J-C, Tsai C-C. Students’ context-specific epistemic justifications, prior knowledge, engagement, and socioscientific reasoning in a mobile augmented reality learning environment. J Sci Educ Technol. 2020 June;29(3):399–408. doi: 10.1007/s10956-020-09825-9

[39] Shela V, Ramayah T, Aravindan KL, et al. Run! this road has no ending! A systematic review of PLS-SEM application in strategic management research among developing nations. Heliyon. 2023 Dec;9(12):e22476. doi: 10.1016/j.heliyon.2023.e22476 38125546 PMC10730991

[40] Chin WW. The partial least squares approach for structural equation modeling In: Modern methods for business research, in methodology for business and management. Mahwah, NJ, US: Lawrence Erlbaum Associates Publishers; 1998. pp. 295–336.

[41] Henseler J, Sarstedt M. Goodness-of-fit indices for partial least squares path modeling. Comput Stat. 2013 Apr;28(2):565–580. doi: 10.1007/s00180-012-0317-1

[42] Medina MS, Castleberry AN, Persky AM. Strategies for improving learner metacognition in health professional education. Am J Pharm Educ. 2017 May;81(4):78. doi: 10.5688/ajpe81478 28630519 PMC5468716

[43] Schellings G. Applying learning strategy questionnaires: problems and possibilities. Metacognition Learn. 2011 Aug;6(2):91–109. doi: 10.1007/s11409-011-9069-5

[44] Howard BC, McGee S, Shia R, et al. Metacognitive self-regulation and problem-solving: expanding the theory base through factor analysis. Expanding the Theory Base through Factor Analysis. 2000 Apr Accessed: Jan. 05, 2026. [Online]. Available: https://eric.ed.gov/?id=ED470973.

[45] Wang C-Y. Exploring general versus task-specific assessments of metacognition in university chemistry students: a Multitrait–Multimethod analysis. Res Sci Educ. 2015 Aug;45(4):555–579. doi: 10.1007/s11165-014-9436-8

[46] Hennrikus EF, Skolka MP, Hennrikus N. Applying metacognition through patient encounters and illness scripts to create a conceptual framework for basic science integration, storage, and retrieval. J Med Educ Curric Dev. 2018 Jan;5:2382120518777770. doi: 10.1177/2382120518777770 29845119 PMC5967154

[47] Michalsky T. Metacognitive scaffolding for preservice teachers’ self-regulated design of higher order thinking tasks. Heliyon. 2024 Jan;10(2):e24280. doi: 10.1016/j.heliyon.2024.e24280 38293459 PMC10827503

[48] Versteeg M, Bressers G, Wijnen-Meijer M, et al. What were you thinking? Medical students’ metacognition and perceptions of self-regulated learning. Teach Learn Med. 2021 Oct;33(5):473–482. doi: 10.1080/10401334.2021.1889559 33722115

[49] Merkebu J, Veen M, Hosseini S, et al. The case for metacognitive reflection: a theory integrative review with implications for medical education. Adv Health Sci Educ. 2024 Sept;29(4):1481–1500. doi: 10.1007/s10459-023-10310-2 PMC11368986 38345690

[50] Eilam B, Aharon I. Students’ planning in the process of self-regulated learning. Contemp Educ Psychol. 2003 July;28(3):304–334. doi: 10.1016/S0361-476X(02)00042-5

[51] Li S, Lajoie SP. Cognitive engagement in self-regulated learning: an integrative model. Eur J Psychol Educ. 2022 Sept;37(3):833–852. doi: 10.1007/s10212-021-00565-x

